# Novel stereological method for estimation of cell counts in 3D collagen scaffolds

**DOI:** 10.1038/s41598-023-35162-z

**Published:** 2023-05-17

**Authors:** Anna Zavadakova, Lucie Vistejnova, Tereza Belinova, Filip Tichanek, Dagmar Bilikova, Peter R. Mouton

**Affiliations:** 1 Biomedical Center, Faculty of Medicine in Pilsen, Charles University, Alej Svobody 76, Pilsen, Czech Republic; 2 Department of Histology and Embryology, Faculty of Medicine in Pilsen, Charles University, Alej Svobody 76, Pilsen, Czech Republic; 3 Imaging and Optics Facility, Institute of Science and Technology Austria, Am Campus 1, Klosterneuburg, Austria; 4 Department of Pathological Physiology, Faculty of Medicine in Pilsen, Charles University, Alej Svobody 76, Pilsen, Czech Republic; 5 Department of Computer Sciences and Engineering, College of Engineering, University of South Florida, 4202 E Fowler Ave, Tampa, FL USA; 6 SRC Biosciences, 1810 W. Kennedy Blvd, Tampa, FL USA

**Keywords:** Cell growth, Cellular imaging, Cell biology, Medical research

## Abstract

Current methods for assessing cell proliferation in 3D scaffolds rely on changes in metabolic activity or total DNA, however, direct quantification of cell number in 3D scaffolds remains a challenge. To address this issue, we developed an unbiased stereology approach that uses systematic-random sampling and thin focal-plane optical sectioning of the scaffolds followed by estimation of total cell number (*StereoCount*). This approach was validated against an indirect method for measuring the total DNA (*DNA content)*; and the *Bürker* counting chamber, the current reference method for quantifying cell number. We assessed the total cell number for cell seeding density (cells per unit volume) across four values and compared the methods in terms of accuracy, ease-of-use and time demands. The accuracy of *StereoCount* markedly outperformed the *DNA content* for cases with ~ 10,000 and ~ 125,000 cells/scaffold. For cases with ~ 250,000 and ~ 375,000 cells/scaffold both *StereoCount* and *DNA content* showed lower accuracy than the *Bürker* but did not differ from each other. In terms of ease-of-use, there was a strong advantage for the *StereoCount* due to output in terms of absolute cell numbers along with the possibility for an overview of cell distribution and future use of automation for high throughput analysis. Taking together, the *StereoCount* method is an efficient approach for direct cell quantification in 3D collagen scaffolds. Its major benefit is that automated *StereoCount* could accelerate research using 3D scaffolds focused on drug discovery for a wide variety of human diseases.

## Introduction

Quantification of cell number in a defined volume is an essential metric for the worldwide communities of bioscientists and pre-clinical researchers. In disciplines involving dispersion of cells into 3D volumes, e.g. 3D collagen scaffolds, reliable estimates of cells are required for launch of experiments, i.e. seeding of cells and assessing cell death and proliferation over time. Enumerating cell numbers in these cases is limited to two major approaches: The *Bürker* chamber for direct cell counts from homogenous cell suspensions (liquid phase); and fluorescence-based approaches for total DNA estimation, e.g., CyQuant®^1,2^. The drawbacks of the total DNA approach are cell lysis from sample pre-processing such as scaffold digestion by collagenase and need for a calibration curve for each experiment to ensure correct read-out and correlations between linearity of the signal intensity to the cell number. Thus, there is no direct quantitative method for estimation of cell counts in 3D scaffolds.

**Figure 1:**
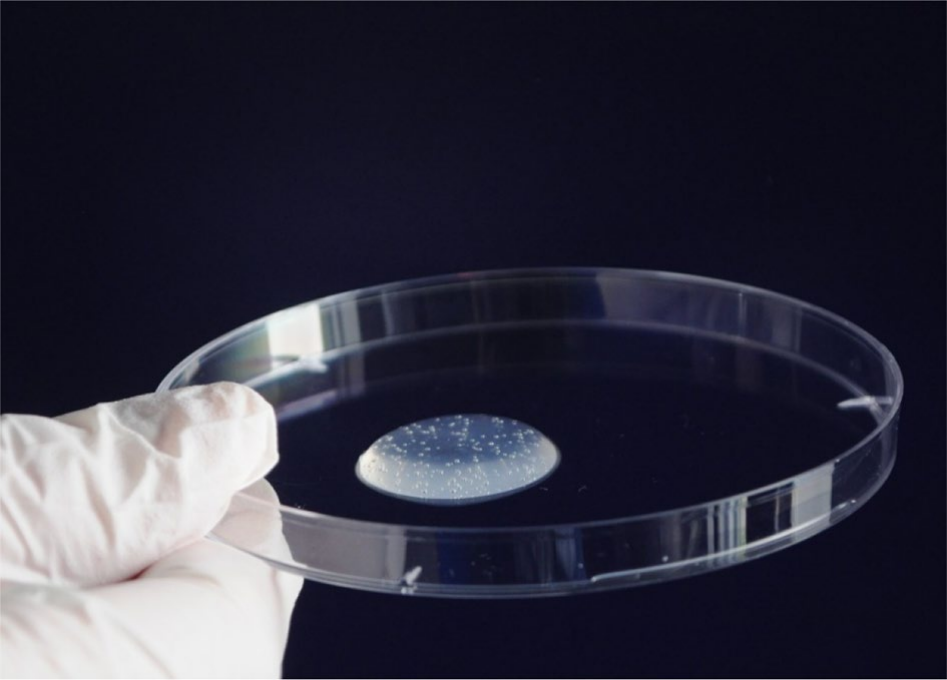
3D collagen scaffold seeded with cells and used for the quantification of cell counts.

To address this issue, we introduce *StereoCount™*, a novel stereology-based method for direct quantification of absolute cell counts in 3D collagen scaffolds (Fig. 1).

**Figure 2:**
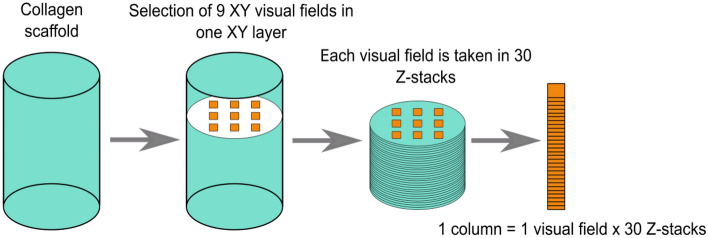
*StereoCount* sampling procedure during microscopy. The three dimensional (3D) collagen scaffolds are sampled into 9 XY visual fields and 30 Z-stacks. Cell count is determined for each column using thin focal-plane optical scanning (optical disector method). The cell count from columns is recalculated to the whole volume of collagen scaffold.

We demonstrate the approach using fluorescence microscopy followed by an optical sectioning and image analysis, as detailed in Materials & Methods and Fig. 2. Second, to avoid bias introduced by manual counting and improve throughput, we introduce an automated workflow using FIJI-ImageJ^3^ that uses two macro scripts and an intermediate, shallow-machine-learning (NL)-based step. Among the benefits are the method does not require scaffold digestion or microtome sectioning and, as a microscope-based approach, provides an overview of cell distribution in the scaffold as additional information. Here we compare and contrast *StereoCount* with *DNA content* method based on fluorescence detection of total DNA; and cell counts using the counting chamber method (*Bürker*), a generally reliable method for cell quantification.

**Figure 3:**
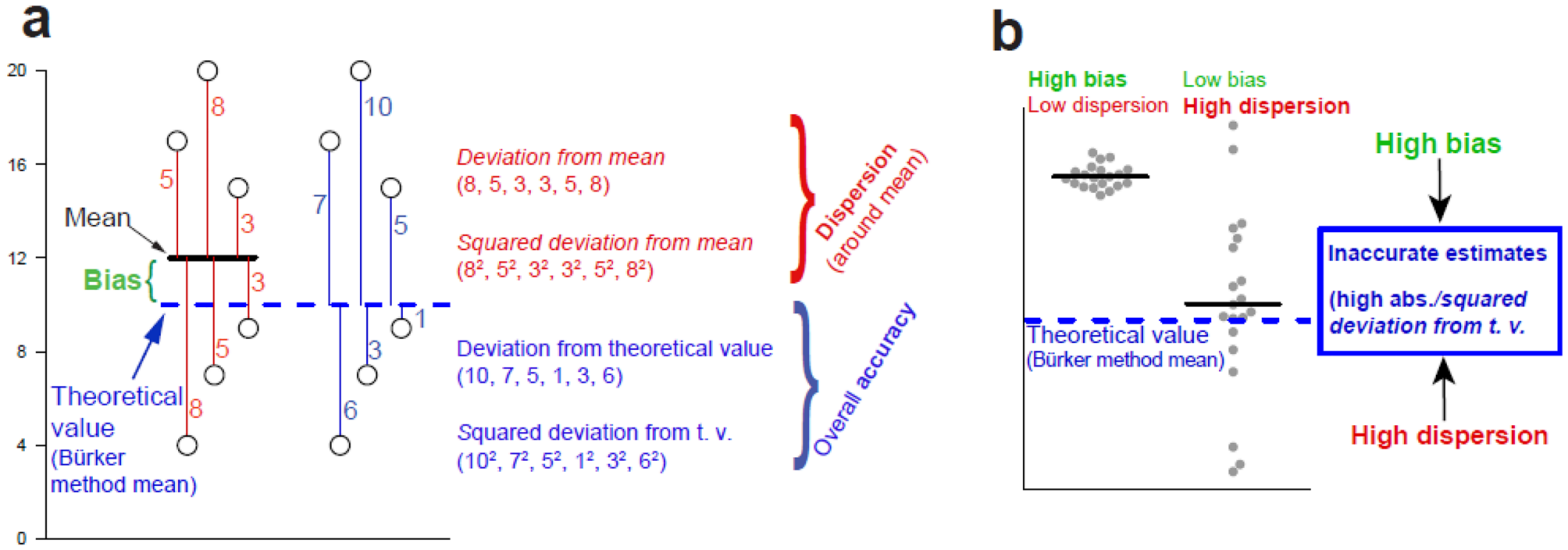
Indicators of the methods’ accuracy. These include ***bias*** (systematic under- or over-estimation of the cell counts relative to *Bürker* method mean), indicators reflecting dispersion around mean (***absolute***/***squared deviation from mean*** estimate of given method) and finally indicators of overall accuracy (***absolute***/***squared deviation from theoretical value*** [t.v.]) reflecting both the bias as well as dispersion (**a**). Both high dispersion as well as bias collectively decreases accuracy of the methods (**b**).

For validation purposes, we compare the accuracy, ease-of-use, equipment requirements and time demands for sample preparation of the cell counts for the cell seeding density (cells per unit volume) across four values estimated by the three methods—*StereoCount*, *DNA content* and *Bürker* (reference standard). Accuracy of the methods are evaluated by these indicators (Fig. 3): (i) **bias** (systematic under- or over-estimation of the cell numbers relative to *Bürker* method mean), (ii) **dispersion** of the estimates (absolute/squared deviation from mean estimate of the given method, in other words how the data are close together) and (iii) **overall accuracy** (absolute/squared deviation from the theoretical value [i.e. the mean estimate from *Bürker* method], in other words the bias and dispersion taken into account mutually).

## Results

### Accuracy comparison

The *Bürker* method assessed the total cell count in the cell suspensions prior to infusion into the 3D collagen scaffolds. Four concentrations of cell suspensions (10,000; 125,000; 250,000 and 375,000 cells) were dispersed in the 3D scaffolds. Two methods (*StereoCount* and *DNA content*) were used to estimate total cell number one day after cell seeding from distinct scaffolds.

**Figure 4:**
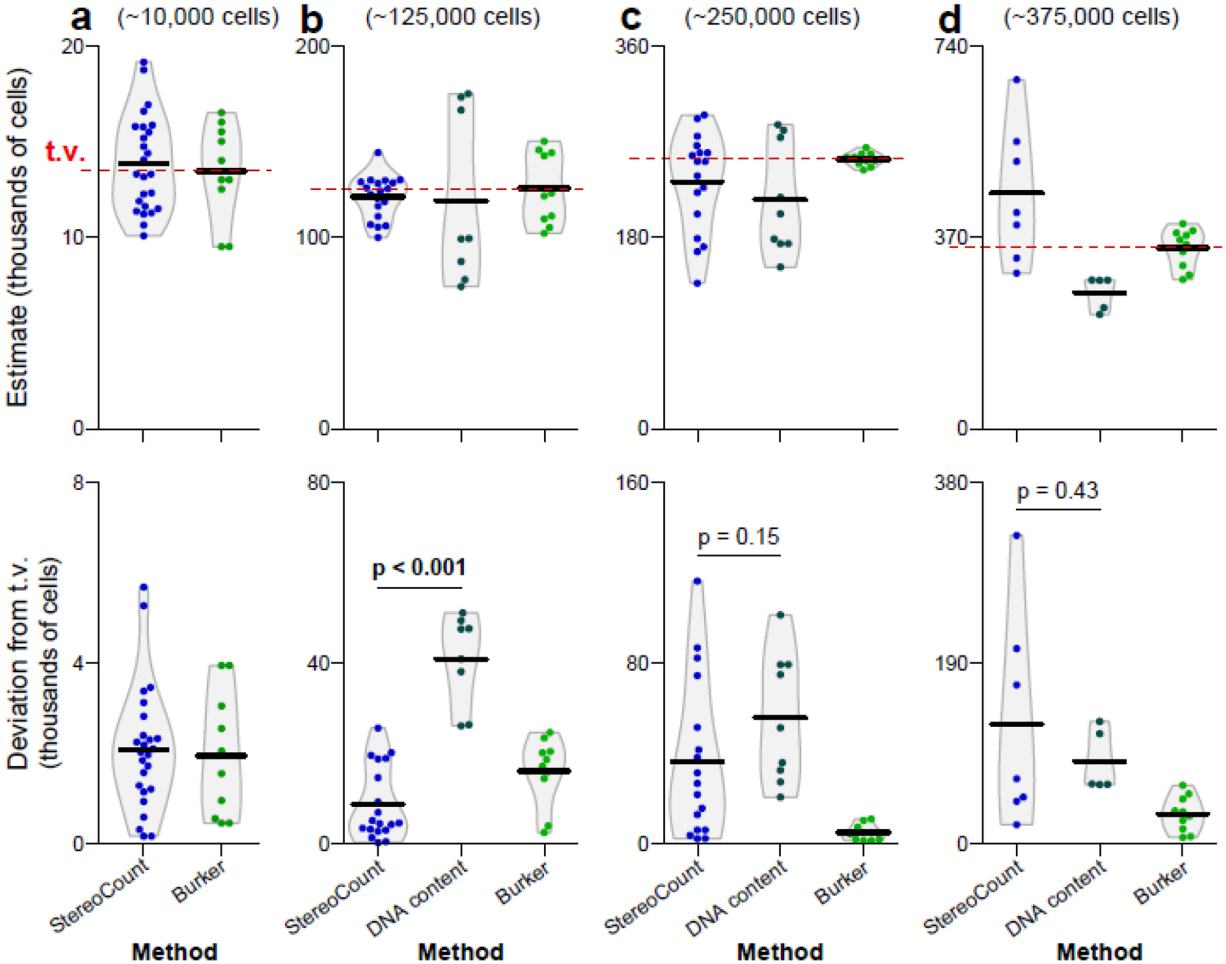
Determined numbers of cells in 3D scaffolds with the cell seeding density across four values (**a**) 10,000, (**b**) 125,000, (**c**) 250,000 and (**d**) 375,000 cells/scaffold (upper part) and *deviation from theoretical value* (t. v., defined as *Bürker* method mean; shown in red dashed line), both showing in thousands of cells. Black horizontal line shows mean.

Accuracy of *StereoCount* and *DNA content* in 3D collagen scaffolds were compared to the counts in the cell suspensions by the *Bürker* method (Supplementary Table S1). Comparisons were done using generalized least squares (GLS) method as shown in Fig. 4 and Table 1. The datasets generated during the current study are available from the corresponding author on reasonable request.

**Table 1:** Results of generalized least squares (GLS) analysis (allowing group-specific variances), comparing the accuracy of the different methods in terms of *bias*, dispersion (*deviation from* [group] *mean*) and overall accuracy (*deviation from theoretical value*) separately for the four concentrations of cells seeded into scaffolds (a–d).

	Bias				Deviation from mean				Deviation from theoretical value			
	β	CI-L	CI-U	*P*	β	CI-L	CI-U	*P*	β	CI-L	CI-U	*P*
[a] 13,450 cells												
Reference (Burker)	0.0	− 1.5	1.5		2.0	1.1	2.8		2.0	1.1	2.8	
StereoCount vs. Burker	0.4	− 1.4	2.2	0.67	0.2	− 0.8	1.1	0.76	0.1	− 0.9	1.2	0.79
[b] 125,400 cells												
Reference (Burker)	0.0	− 11.5	11.5		16.1	11.4	20.7		16.1	11.4	20.7	
StereoCount vs. Burker	− 4.4	− 16.9	8.2	0.49	− 7.2	− 12.6	− 1.7	**0.021**	− 7.4	− 13.3	− 1.5	**0.028**
DNA content vs. Burker	− 6.4	− 39.2	26.4	0.70	23.2	12.4	34.1	**0.001**	24.8	16.5	33.2	** < 0.001**
StereoCount vs. DNA content					− 30.4	− 40.6	− 20.2	** < 0.001**	− 32.2	− 40.1	− 24.3	** < 0.001**
[c] 253,200 cells												
Reference (Burker)	0.0	− 3.8	3.8		4.8	2.6	7.0		4.8	2.6	7.0	
StereoCount vs. Burker	− 21.2	− 43.5	1.2	0.087	33.0	20.9	45.1	** < 0.001**	31.6	14.9	48.3	** < 0.001**
DNA content vs. Burker	− 38.0	− 72.2	− 3.8	**0.044**	38.8	23.2	54.5	** < 0.001**	51.0	32.2	69.9	** < 0.001**
StereoCount vs. DNA content					− 5.8	− 25.4	13.7	0.565	− 19.4	− 44.4	5.6	0.148
[d] 349,200 cells												
Reference (Burker)	0.0	− 23.3	23.3		31.0	19.5	42.5		31.0	19.5	42.5	
StereoCount vs. Burker	106	4.5	207.3	0.057	77.5	29.1	125.8	**0.002**	94.5	11.0	178.0	**0.018**
DNA content vs. Burker	− 86	− 123.4	− 49.1	**0.003**	− 2.3	− 15.7	11.2	0.743	55.3	24.1	86.5	**0.003**
StereoCount vs. DNA content					79.8	32.2	127.3	**0.005**	39.2	− 48.4	126.8	0.430

β = estimated effect. CI-L/CI-U = lower and upper bounds of the 95% confidence intervals for the β, based on the GLS. *P* = *p*-value from permutational t-test (two-sided). Numbers for β and CI-L/U are shown in thousands. The first row shows data for results of the reference method (*Bürker*) whereas the other rows show comparison between two methods of estimation. For example, for table part ‘b’, *deviation from mean*: β in the first row is 16.1, meaning that the deviation from mean for the *Bürker* method was 16,100 in average, for the scaffold of 125,000 cells. The deviation from the mean for *StereoCount* was smaller by 7200 cells [CI 1700; 12,600] compared to *Bürker*, and this difference was statistically significant (*p* = 0.021).

Significant values are in [bold].

The reference values were based on mean estimates from *Bürker* method. The *Bürker* counts were 13,450 ± 2477 cells (mean ± SD) for the concentration of 10,000 cells/scaffold (Fig. 4a, Table 1a). Estimation by *StereoCount* showed no difference from *Bürker* in terms of accuracy or dispersion (variation) of the estimates. In contrast, the *DNA content* method lacked the sensitivity for estimation at this low cell density.

For the scaffolds infused with about 125,000 cells, *Bürker* estimated 125,400 ± 18,536 cells (Fig. 4b, Table 1b) and neither *StereoCount*, nor *DNA content* showed differences from *Bürker*. However, *StereoCount* method closely matched the reference method (*Bürker)* in terms of overall accuracy and dispersion. In contrast, *DNA content* showed a statistically greater difference of ~ 30,400 cells (95% CI [20,200; 40,700], *p* < 0.001) from the mean estimate of the reference method compared with *StereoCount* and higher deviation from the theoretical value by 32,200 cells (CI [24,300; 40,100], *p* = 0.001). Interestingly, the results from *StereoCount* showed even smaller dispersion than *Bürker* (reference method) as the deviation from mean was smaller by 7200 cells (CI [1700; 12,600], *p* = 0.021), i.e., closer to the initial number of cells seeded (125,000 cells/scaffold).

For the scaffolds of 250,000 cells, *Bürker* estimated 253,200 ± 6197 cells (Fig. 4c, Table 1c). Both *StereoCount* and *DNA content* methods showed differences from these reference values in terms of overall accuracy and dispersion. Specifically, the deviation from *Bürker* was higher for *StereoCount* by 31,600 cells [CI 14,900; 48,300] and for *DNA content* by 51,000 cells [CI 32,200; 69,900]. Although only *DNA content* underestimated the cell count by 38,000 cells [CI -72,200; -3800], *p* = 0.044), the dispersion and overall accuracy of both *StereoCount* and *DNA content* were not different from each other (*p* > 0.05).

Finally, for the highest density cell counts in the scaffolds (375,000 cells), *Bürker* estimated 349,200 ± 37,542 cells (Fig. 4d, Table 1d). Both *StereoCount* and *DNA content* differ from these reference values and did not differ from each other in terms of overall accuracy. However, *DNA content* tended to produce lower dispersion of the estimates than *StereoCount:* whereas the deviation from mean was higher in *StereoCount* by 77,500 cells [29,100; 125,800] compared to *Bürker* (*p* = 0.005)*, DNA content* showed comparable deviation from the mean as *Bürker* (the difference of 2300 cells [-11,200; 15,700] *p* = 0.743)*.* Although *DNA content* showed bias (underestimation) of -86,000 cells [CI -123,400; -49,100] (*p* = 0.003), the method tended toward lower dispersion of the estimates than *StereoCount* (*DNA content* deviation from mean was smaller by 79,800 cells [32,200; 127,300] compared to *StereoCount* (*p* = 0.005). Supplementary Table S2 provide further results of analyses of squared deviation from mean and squared deviation from theoretical value.

### Ease-of-use, equipment required and time demands for sample preparation

In addition to accuracy relative to the reference method, other factors for consideration include ease-of-use, costs, equipment requirements and time demands for each method.

**Figure 5:**
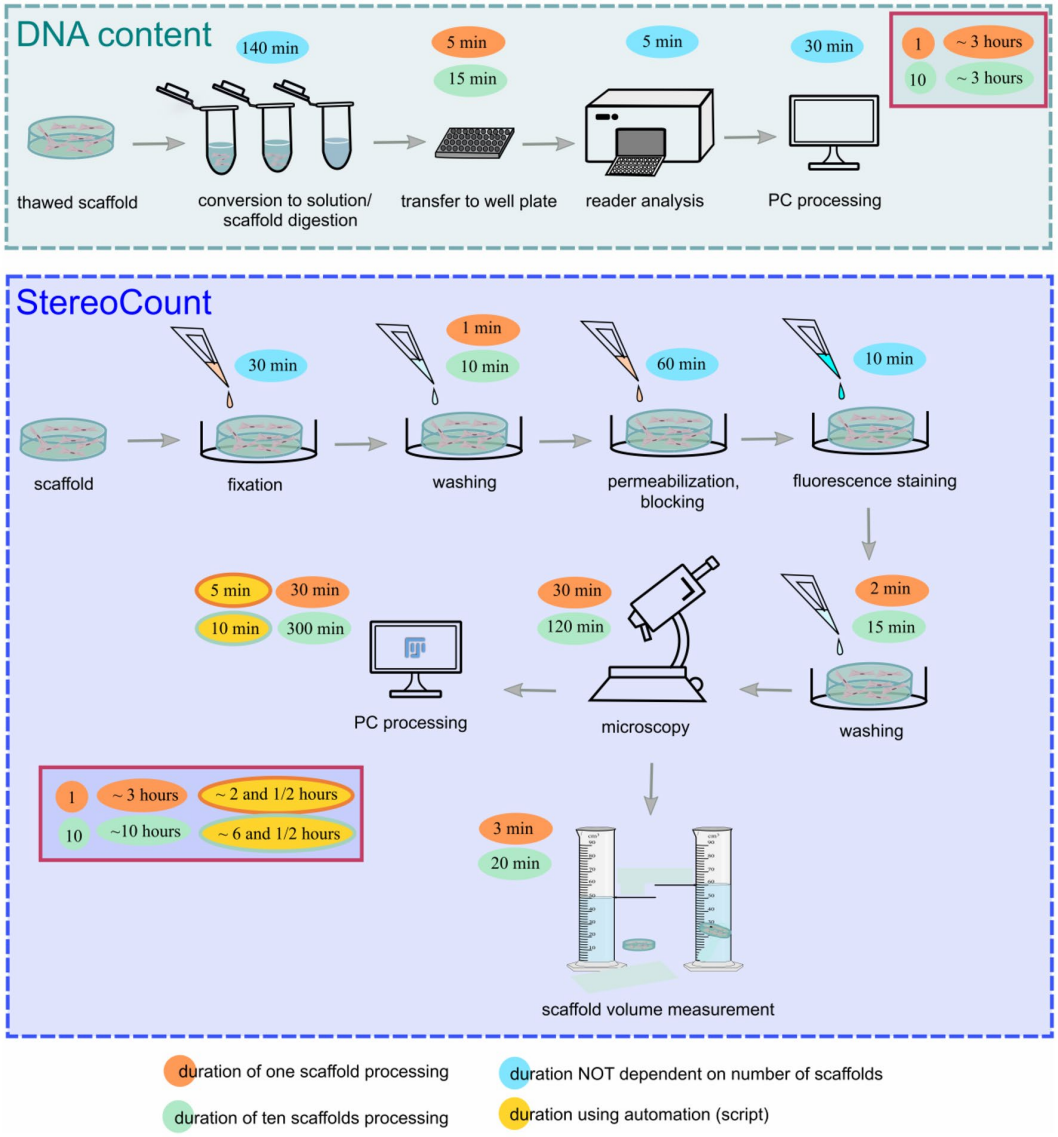
Scheme of *DNA content* (upper panel) and *StereoCount* (lower panel) processes with time demands of each approach in a step-by-step manner. Process duration is not dependent on the sample size (blue). Process duration of one scaffold analysis (orange). Process duration of ten scaffolds analyses (green). Process duration when automation (script) is used for data processing (yellow). Total time demands for one or ten samples for each method are given in red frames.

*DNA content* is a widely used fluorescence-based method that is rapid and easy to perform. Microplate reader with fluorescence signal detection is needed. The approach is well-suited to multiple-well format, whereas more scaffolds require similar processing time as cell estimation for a single scaffold. For example, analysis of one or ten scaffolds takes about three hours (Fig. 5). However, due to the requirement of scaffold digestion, information about the cell distribution is lost and no further processing or analysis is possible. Another drawback is the output is not cell count but rather a relative fluorescence unit of total DNA per well (Table 2) which requires a reference standard curve for converting sample fluorescence values into cell numbers.

**Table 2:** Comparison of advantages, disadvantages and equipment required for the different cell count methods.

Method	Advantages	Disadvantages	Final output	Laboratory equipment
DNA content	Multiple-well format Fast assay Easy to perform	Requires scaffold digestion Indirect measurement	Relative fluorescence unit of DNA content per well	Microplate reader
StereoCount	absolute cell count Does not require scaffold digestion or sectioning Provides information about cell distribution	Time-consuming (manual format)	Cell number per whole scaffold	Fluorescence microscope

The novel *StereoCount* approach proposed here is based on unbiased stereology principles for cell number estimation in 3D collagen scaffolds. Fluorescence microscope is required for the image acquisition. Among the advantages of *StereoCount* are the approach (i) provides an absolute count of cells per scaffold (Table 2); and (ii) does not require scaffold digestion, thereby conserving information about cell distributions in 3D. In terms of time, *StereoCount* analysis of one scaffold and ten scaffolds takes about 3 and 10 h, respectively. It is expected that automation will reduce the time of data processing to approximately 10 min or less depending on computer specifications (Fig. 5). The script for the high throughput analysis can be found in Supplementary Macro S1.

## Discussion

Here we review several methods for assessing cell proliferation in 3D scaffolds. One common strategy for estimating cell proliferation is based on the conversion of various substrates by cellular enzymes to highly fluorescent products, e.g., MTT^4,5^, WST-1^6^, WST-8/CCK-8, CCK-8^7^ and AlamarBlue assay^8^. Since these methods depend on the activity of mitochondrial enzymes, the results reflect the metabolic activity of the cells, rather than the single cell count per se. Moreover, this process loses sensitivity as metabolic activity changes such as in the cases of senescent or slowly grown cells^9^.

Methods based on labeling of DNA content, e.g., CyQuant^1,2^, are independent of metabolic activity of the cells. However, these approaches only estimate the relative fluorescence unit, which requires DNA standard curves for conversion to DNA content per cell, i.e. a standard curve generated from the DNA content for a known number of cells seeded on a plate. Another complication is that, compared to usual 2D cultures, the estimation in 3D scaffolds requires collagen hydrogel digestion prior to detection of a fluorescent signal from the labeled cellular DNA. Although this strategy is common, the approach only provides information about the amount of DNA present, and gives no information about the absolute cell count or the cell distribution throughout the 3D scaffolds.

To address this need, we developed a novel stereology-based strategy, *StereoCount*, for quantifying the cell number in a 3D scaffold, without need for digestion or calibration curves for each experiment. We assessed *StereoCount* in comparison with a current and commonly used method, *DNA content*, using the counting chamber (*Bürker*) as a reference method. Finally, as an extension we developed an automated workflow (pipeline) to avoid the introduction of subjectivity errors from manual counting. Efficiency of the automated workflow could be potentially improved by changing the macro scripts, and further automated by combining all of the steps into one macro or switching to different platforms for each step. Our easy-to-apply workflow provides for reliable results with identical error applied to each image, avoiding inter-rater error caused by different investigators performing each task in a manual manner.

Our data shows that for low cell numbers (10,000 cells/scaffold) *DNA content* is not usable due to the background fluorescence signal exceeding the signal from the florescent-stained DNA. For low cell density, therefore, *StereoCount* is the preferred choice since the approach is based on simple counting of cells in a known fraction of the total volume. For higher cell numbers (125,000 and 250,000 cells/scaffold), both *StereoCount* and *DNA content* provide useful results, though *StereoCount* more closely reflect the loading number of cells using the reference *Bürker* method. For higher cell density (250,000 cells/scaffold and higher) both *DNA content* and *StereoCount* are the similarly effective. The limitation for *StereoCount* is the extra time and care required for avoiding overlapping cells as the cell density increases. Lack of care to this issue could lead to a systematic error (bias) that favors undercounts as the cell density increases. Similarly, our results show underestimation of the cell counts by *DNA content* as a direct function of increasing cell density, suggesting a bias-correction may be needed for this approach as well. The difference is that for *StereoCount* these undercounts may be avoided by careful attention at high cell density, whereas this strategy is not available for the *DNA content* method.

Finally, we consider feasibility, material, machine requirements, and time demands of the *StereoCount* and *DNA content* methods. A strong advantage of *DNA content* is multiple-well format option that is less time-consuming method as compared to *StereoCount*, although the data processing (especially the multiple sample analysis) is substantially shortened by the use of an automated workflow for *StereoCount*. However, the faster analysis of data for *DNA content* is off-set by the need for building a calibration curve to estimate cell count from relative fluorescent units for each experiment. The strongest advantage of *StereoCount* is the ability for direct cell counts and visualization of cell distribution in 3D using simple microscopic images, without the need for scaffold digestion or microtome sectioning. Though the *StereoCount* approach is illustrated here using fluorescent-stained cells, the same approach is applicable to differentiate between live and dead cells when DAPI and Calcein-AM staining is combined, as well as approaches that use immunohistochemical staining of cells in the 3D scaffolds.

## Conclusions

Although hydrogel scaffolds are nowadays an ordinary type of 3D cell culture, there remains a large and growing need for quantitative tools to assess cell proliferation and other parameters following different experimental manipulations. Our novel stereology-based freeware approach, *StereoCount*, uses optical sectioning of stained cells in non-digested collagen hydrogels with faster throughput using the provided automated workflow. We report that *StereoCount* is more suitable for lower cell density (~ 10,000 and ~ 125,000 cells/scaffold), where the *DNA content* method lacked the sensitivity for estimation of such low cell density, while *DNA content* by the fluorescence-based indirect method may be preferable in for higher cell density (~ 250,000 and ~ 375,000 cells/scaffold).

## Materials and methods

### Cell isolation and culture

Normal human dermal fibroblasts (NHDF) were isolated from skin following plastic surgery interventions after approval by the local ethics committee of the University Hospital in Pilsen, E. Benese 13, 305 99 Pilsen, Czech Republic, decision of November 5th, 2015. The guidelines in the Declaration of Helsinki were followed. All donors gave written informed consent before intervention. The samples were washed by Hank’s balanced salt solution (HBSS) (Merck KGaA, Darmstadt, Germany) containing penicillin (100 U/ml)/streptomycin (0.1 mg/ml) (Biochrom, Cambridge, UK) and gentamicin (50 μg/ml) (Biochrom). The samples were cut and digested overnight at 37 °C in HBSS containing collagenase type I (100 U/ml, Merck KGaA). On the following day, the suspension was shaken intensively and filtered through 100 µm nylon cell strainer (Falcon™, Thermo Fisher Scientific, Waltham, Massachusetts). The cell suspension was then transferred to a cultivation flask (TPP) containing low glucose Dulbecco’s Modified Eagle’s Medium (DMEM) (Merck KGaA), 10% heat-inactivated fetal bovine serum (FBS) (Merck KGaA), penicillin (100 U/ml)/streptomycin (0.1 mg/ml) (Biochrom), 0.5% L-glutamin (Biosera, Nuaille, France) and 1.0% non-essential amino acids (Biosera). The NHDF were cultured at 37 °C, 5% CO_2_ up to 80% confluence and then passaged. Only those NHDF from the 2th–5th passages were used in the experiments.

### Isolation of rat tail collagen type I

The type I collagen used for preparing the 3D collagen scaffold was isolated from rat tails. The collection of rat tails for collagen isolation was approved by the Animal Welfare Advisory Committee of the Ministry of Education, Youth and Sports of the Czech Republic (approval ID MSMT-249/2017-2) and conducted under the supervision of the Animal Welfare Advisory Committee of the Charles University Faculty of Medicine in Pilsen following the technological, hygienic, welfare and ethical standards given by Directive 2010/63/EU and FELASA Guidelines and Recommendations (the workplace accreditation number 4891/2015-MZE-17214). The strain of rats was Wistar, the gender was male, and the age was 6–7 months. The rats were used in another independent study without any effect on the tails. Thus the tails were collected after animals sacrifice approved by the protocol above (approval ID MSMT-249/2017-2). The study was carried out in compliance with the ARRIVE guidelines. The tendons were removed from the rat tails and maintained 3 × 24 h in PBS on a laboratory shaker at room temperature. PBS was exchanged every single day. Afterwards, the procedure was repeated with citrate buffer (0.08 M, pH 3.7). Subsequently, the tendons were digested in 0.1 M acetic acid for 48 h at 4 °C. The collagen digested in acetic acid was homogenized with blender and the suspension was ultracentrifuged at the maximum speed (approx. 46,000× g) for 1 h in order to remove the tissue debris. The supernatant containing the collagen type I was lyophilized and stored in -20 °C. The collagen solution for preparation of the 3D collagen scaffold was prepared by dissolving the lyophilized collagen in 0.02 M acetic acid at 4 °C at least 5 days to a concentration of 5 mg/ml and was stored at 4 °C as a stock solution for maximal 1 month.

### Preparation and culture of cell-seeded collagen scaffolds

3D collagen scaffolds containing cells were prepared as follows. 600 µl of collagen stock solution (5 mg/ml) was mixed properly with 290 µl of culture medium, 10 µl of sodium bicarbonate (Merck KGaA) and 100 µl of cell suspension in medium on ice. 0.5 ml of collagen suspension with cells was plated into the 24-well plate. The final collagen concentration was 3 mg/ml and the final cell counts seeded were 10,000; 125,000; 250,000 and 375,000 cells per 0.5 ml. 0.5 ml of collagen suspension with homogeneously distributed cells was plated into the 24-well plate. The polymerization occurred at 37 °C, 5% CO_2_ and humidified atmosphere after 20 min. The change in pH from acidic to neutral and 37 °C caused the collagen to polymerize and self-assembly into a gel. After collagen polymerization 0.5 ml of culture medium was carefully added on the top of the hydrogels. The culture medium was exchanged 2–3 times a week.

### Cell number estimation by stereology (StereoCount)

#### Cell staining and collagen scaffold optical sectioning by microscopy

The day after their preparation the cell-seeded collagen scaffolds (N = 25 for 10,000 cells/scaffold; N = 19 for 125,000 cells/scaffold; N = 17 for 250,000 cells/scaffold; N = 7 for 375,000 cells/scaffold) were fixed with 4% paraformaldehyde (Merck KGaA) for 30 min. After washing with PBS the collagen scaffolds were maintained in 0.2% Triton X-100 (Merck KGaA) for 30 min followed by 30 min in 5% BSA (Merck KGaA). Nuclei of NHDF in collagen scaffolds were stained by DAPI (1:1000) for 10 min in the dark at room temperature. The process of optical sectioning of cell-seeded collagen scaffolds was as follow. Nine Z-axis columns were located through the XYZ-axes of each collagen scaffold (Fig. 2). The columns included 30 separated Z stacks with 0.02 mm distance between each stack giving a total volume of each column of 1.4 mm^3^ = 1.4 μl. Thus, the total volume of all 9 columns was 12.6 mm^3^. The mean coefficient of variance (CV) for the sampling of 9 columns was in the range of 9–20% and is comparable with CV of *DNA content* and *Bürker* methods (Supplementary Table S1) and with other studies^10,11^. 2D images of each section were taken by Olympus UPlanFL N 10x/0.30 objective and the pictures of columns were saved as image sequence (*.stk file) by VisiView® software. The number of cells was recalculated to the whole collagen scaffold volume.

#### Cell number estimation by image analysis

Cell numbers were counted within columns using FIJI-ImageJ software (National Institute of Health, Bethesda, USA) and cell number in total volume of a collagen scaffold was estimated from each column using the optical disector principle^12^. Further details on the schema can be found in Supplementary Fig. S1.

#### Automated workflow for cell number estimation by image analysis

**Figure 6:**
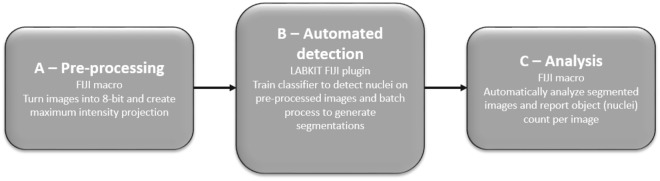
Scheme of an automated workflow which consists of two macro scripts (**a**,**c**) and an intermediate (**b**), shallow-machine-learning-based step.

After acquisition, we first batch process images to make them easier to work with. First provided short macro (Supplementary Macro S1) automatically converts the acquired images to 8-bit images and maximum intensity Z projection (Fig. 6a). The following, intermediate, step requires significant user input in the beginning (Fig. 6b). Herein we used a LABKIT^13^ plugin in FIJI which uses a shallow machine learning approach to segment images, based on training. To generate a training dataset, we concatenated six sample images (pre-processed with step A of the workflow) from different cell seeding density. Afterwards a user-induced training was applied to generate a classifier. This classifier was used to batch process all pre-processed images from step A and generate segmentations. These segmentations were then analyzed in step C (Fig. 6c) of the workflow. We applied additional short macro (Supplementary Macro S2) to batch-analyze the masks and report count of detected objects per image. We apply the macros for free use without any guarantees and maintains.

#### Estimation of collagen scaffold volume

Collagen scaffold volume was estimated according to Archimedes´ principle. 5-ml measuring cylinder with 0.1 ml marked lines was filled with precise 3 ml of PBS. Each collagen scaffold was put into the cylinder with PBS and the buoyed volume of PBS was read on the cylinder scale. The increased volume in the cylinder corresponded to the volume of the collagen scaffold.

#### Cell number calculation

The cell count in acquired image sequences (XYZ column) was recalculated to the whole cell-seeded collagen scaffold, i.e. for estimation of cell number in one collagen scaffold, cell count from 9 image sequences (i.e. 9 columns) were recalculated to the total collagen scaffold volume. This calculation was performed from the 9 columns and the mean of the values was considered as a result (total cell count) for one collagen scaffold.

### Cell number estimation by Bürker chamber method (Bürker)

The NHDF-seeded culture dishes were trypsinized and resuspended in fresh culture medium. A portion of the suspension was diluted with Trypan blue (Merck KGaA) for the cell count estimation (five repetitions). The mean estimate of cell number served as an initial cell count for further dilutions.

From the initial cell suspensions 5 × 0.5 ml (containing 10,000; 125,000; 250,000 and 375,000 cells) were prepared. From these 0.5 ml-suspensions the portion was diluted with Trypan blue and the cell number was estimated. This counting was performed in 10 repetitions by two independent observers (N = 10 of each cell seeding density).

### Cell number estimation by total DNA quantification (DNA content)

Cell-loaded collagen scaffolds (N = 4 for 10,000 cells/scaffold; N = 8 for 125,000 cells/scaffold; N = 9 for 250,000 cells/scaffold; N = 5 for 375,000 cells/scaffold) were prepared according to the chapter “Preparation and culture of cell-seeded collagen scaffolds”. The next day, the scaffolds were washed with PBS and placed into − 80 °C. On the day of analysis, the collagen scaffolds were thawed.

Firstly, Proteinase K (0.2 mg/ml; 2.5 DMC-U/ml, Serva, Heidelberg, Germany) in phosphate buffered EDTA (PBE) was added to scaffolds with cells in 1:1 ratio. The samples were then incubated for 90 min at 50 °C. Afterwards, the digested sample fluid was homogenized by pipetting and vortexed. 462 µl of the sample fluid (half of the sample volume) was transferred to fresh tube and 17.6 µl of RNAse A (1 mg/ml, Serva), 1 µl of EDTA (0.5 M, Thermo Fisher Scientific) and 20 µl of NaCl-Tris–EDTA (TE) buffer solution (10.5 mg NaCl per sample diluted in 50 × TE buffer) was added and incubated for 1 h at room temperature. RNAse-processed sample fluid was transferred to black 96-well plate (100 µl/well) and 50 µl of CyQuant solution (Thermo Fisher Scientific) in TE buffer was added (final TE buffer concentration 1 × , CyQuant:TE buffer 1:200). Samples were incubated at room temperature, shielded from light for 10 min and occasionally shaken. Fluorescence signal was measured by microplate reader (Synergy HT, Biotek, Winooski, Vermont) at 520 nm/480 nm and correlated to DNA standard prepared according to CyQuant manufacture brochure.

The DNA content in cell-seeded collagen scaffolds was recalculated to cell number according to cell number calibration curve made from the cells seeded usually on a plate.

### Data processing and statistical analysis

Data were analyzed using R statistical software^14^. R packages ‘beeswarm’^15^ and ‘vioplot’^16^ were used for data visualization.

The accuracies were compared using: (i) ***bias*** from the theoretical values, indicating how much the average estimates from the *StereoCount* and *DNA content* estimators differ from the *Bürker*, reference method. Next, we used two measures reflecting dispersion, ignoring the potential bias and thus enabling meaningful comparison of the *Bürker* vs. other methods. These are (ii) ***deviation from*** [group-specific] ***mean*** and (iii) ***squared deviation from mean***. Finally, two other measures indicate overall accuracy of the methods and reflect both the *bias*, as well as the dispersion: (iv) ***deviation from theoretical value*** and (v) ***squared deviation from the theoretical value*** (Fig. 3).

All these five measures of accuracy were originally compared using generalized least square (GLS), with variance function allowing estimator-specific variance, using ‘nlme’ package^17^. As models of squared deviations exhibited non-normal distribution of standardized residuals (checked visually using histograms and Q-Q plots and via Shapiro–Wilk test), they were re-analyzed using generalized linear models with Gamma distribution and log-link (Gamma GLM).

In each model, one of the five above-mentioned measures represented the outcome (response variable) whereas the type of the method represented a predictor (explaining variable). *P*-value (two-sided) was based on permutational t-test (bias and absolute deviations) or permutational Gamma GLM (squared deviations), according to the script shown previously^18^ using 5000 Monte-Carlo randomizations. The permutation approach was chosen because it estimates statistical significances reliably even if sample sizes are small and assumptions of the fully parametric methods (including normal distribution of residuals) are not met^19^. *P*-values were not corrected for multiple comparisons because we concentrated particularly on the single comparison (*StereoCount* vs. *DNA content*) and 95% confidence intervals for effect size were derived from the GLS and Gamma GLM models.

## Supplementary Information

Supplementary Information 1.

Supplementary Information 2.

Supplementary Information 3.

Supplementary Information 4.

Supplementary Video 1.

## Data Availability

The datasets generated during the current study are available from the corresponding author on reasonable request.
