# Author Correction: Novel stereological method for estimation of cell counts in 3D collagen scaffolds

**DOI:** 10.1038/s41598-023-37265-z

**Published:** 2023-06-21

**Authors:** Anna Zavadakova, Lucie Vistejnova, Tereza Belinova, Filip Tichanek, Dagmar Bilikova, Peter R. Mouton

**Affiliations:** 1 Biomedical Center, Faculty of Medicine in Pilsen, Charles University, Alej Svobody 76, Pilsen, Czech Republic; 2 Department of Histology and Embryology, Faculty of Medicine in Pilsen, Charles University, Alej Svobody 76, Pilsen, Czech Republic; 3 Imaging and Optics Facility, Institute of Science and Technology Austria, Am Campus 1, Klosterneuburg, Austria; 4 Department of Pathological Physiology, Faculty of Medicine in Pilsen, Charles University, Alej Svobody 76, Pilsen, Czech Republic; 5 Department of Computer Sciences and Engineering, College of Engineering, University of South Florida, 4202 E Fowler Ave, Tampa, FL USA; 6 SRC Biosciences, 1810 W. Kennedy Blvd, Tampa, FL USA

Correction to: *Scientific Reports* 10.1038/s41598-023-35162-z, published online 17 May 2023

The original version of this Article contained errors in the Materials and methods section as well as in the Supplementary Information.

In the Materials and methods section, under the subheading ‘Cell number estimation by total DNA quantification (DNA content)’,

“Cell-loaded collagen scaffolds (N = 4 for 10,000 cells/scaffold; N = 8 for 125,000 cells/scaffold; N = 9 for 250,000 cells/scaffold; N = 5 for 375,000 cells/scaffold) were prepared according to 5.3.”

now reads:

“Cell-loaded collagen scaffolds (N = 4 for 10,000 cells/scaffold; N = 8 for 125,000 cells/scaffold; N = 9 for 250,000 cells/scaffold; N = 5 for 375,000 cells/scaffold) were prepared according to the chapter Preparation and culture of cell-seeded collagen scaffolds.”

In Supplementary Information 1, the Supplementary Figure S2 legend has been deleted, which was erroneously present in this file from a previous version.

The original Article and accompanying Supplementary Information 1 have been corrected.

