# Supplementary material for: Novel stereological method for estimation of cell counts in 3D collagen scaffolds
Source: Sci Rep. 2023 May 17;13:7959. doi: 10.1038/s41598-023-35162-z (PMC10192446; doi:10.1038/s41598-023-35162-z)
Supplement: Supplementary file 1 — Supplementary Information 1. [file 41598_2023_35162_MOESM1_ESM.pdf]

Novel stereological method for estimation of cell counts in 3D collagen scaffolds

Anna Zavadakova[1*], Lucie Vistejnova[1,2], Tereza Belinova[1,3], Filip Tichanek[1,4], Dagmar Bilikova[1], Peter R. Mouton[5,6]

[1]Biomedical Center, Faculty of Medicine in Pilsen, Charles University, Alej Svobody 76, Pilsen, Czech Republic

[2]Department of Histology and Embryology, Faculty of Medicine in Pilsen, Charles University, Alej Svobody 76, Pilsen, Czech Republic

[3]Imaging and Optics Facility, Institute of Science and Technology Austria, Am Campus 1, Klosterneuburg, Austria

[4]Department of Pathological Physiology, Medical Faculty in Pilsen, Charles University, Alej Svobody 76, Pilsen, Czech Republic

[5]Department of Computer Sciences and Engineering, College of Engineering, University of South Florida, 4202 E Fowler Ave, Tampa, FL, USA

[6]SRC Biosciences, 1810 W. Kennedy Blvd, Tampa, FL, USA

*corresponding author: anna.zavadakova@gmail.com

Supplementary file

Supplementary Table S1 Primary data obtained from the methods: *StereoCount* (a), *DNA content* (b) and *Bürker* (c) and the coefficients of variation (CV) in %.

	**[a] Cell number per scaffold**				**[b] Cell number per scaffold**				**[c] Cell number in cell suspension**			
	**10,000**	**125,000**	**250,000**	**375,000**	**10,000**	**125,000**	**250,000**	**375,000**	**10,000**	**125,000**	**250,000**	**375,000**
	16,575	99,800	265,970	300,419	-3,291	77,818	280,462	286,939	15,500	123,000	255,000	342,000
	11,483	110,809	237,623	329,474	-8,912	74,224	285,664	287,134	12,500	105,000	243,000	378,000
	12,310	105,869	251,023	673,922	-5,258	87,274	273,691	286,530	9,500	111,000	264,000	382,500
	11,201	129,935	226,577	554,619	-6,291	166,330	217,356	220,619	16,500	109,500	252,000	288,000
	14,031	105,268	294,762	417,344		173,081	173,811	233,585	13,000	102,000	246,000	297,000
	10,071	106,520	256,705	393,607		174,873	201,886		14,000	121,500	252,000	364,500
	11,878	125,782	251,078	516,282		99,327	173,879		13,000	150,000	255,000	396,000
	13,144	128,064	246,479			99,064	151,795		15,000	142,500	249,000	355,500
	11,352	124,159	259,116				178,263		9,500	144,000	258,000	373,500
	10,611	125,219	274,862						16,000	145,500	258,000	315,000
	13,292	116,216	166,366									
	10,629	118,542	136,754									
	15,776	128,765	178,690									
	19,138	128,343	170,870									
	18,728	129,767	201,708									
	15,845	122,284	221,911									
	14,733	120,321	291,335									
	12,246	129,584										
	15,169	144,134										
	11,605											
	11,265											
	14,380											
	15,477											
	16,913											
	15,752											
**CV (%)**	**20**	**9**	**12**	**15**	**--**	**37**	**24**	**13**	**18**	**15**	**2**	**11**

Supplementary Table S2 Results of generalized linear models with Gamma distribution and log-link function (Gamma GLM), comparing the accuracy of the different methods in terms of dispersion (*squared deviation from* [group] *mean*) and overall accuracy (*squared deviation from theoretical value*) separately for the four concentrations of cells seeded into scaffolds (a-d). ß=estimated effect: the first row shows estimates of mean squared deviation for the reference estimator (*Bürker*), and other rows show comparison between different methods of estimation in terms of relative values (ß= 0.5 means that the indicator reach 50% of value compared to the second method of estimation). CI-L/CI-U= lower and upper bounds of the 95% confidence intervals for the ß, based on the Gamma GLM. P= p-value from permutational Gamma GLM (two-sided).

	Squared deviation from mean				*Squared deviation from theoretical value*			
**[a] 13,450 cells**	**ß**	**CI-L**	**CI-U**	**P**	**ß**	**CI-L**	**CI-U**	**P**
Reference (Burker)	exp(15)				exp(15)			
StereoCount vs. Burker	1.09	0.44	2.43	0.89	1.12	0.42	2.66	0.88
**[b] 125,400 cells**	**ß**	**CI-L**	**CI-U**	**P**	**ß**	**CI-L**	**CI-U**	**P**
Reference (Burker)	exp(20)				exp(20)			
StereoCount vs. Burker	0.38	0.17	0.83	**0.011**	0.45	0.18	1.01	**0.038**
DNA content vs. Burker	5.6	2.1	15.1	**0.001**	5.7	2.0	16.6	**<0.001**
StereoCount vs. DNA content	0.07	0.03	0.17	**<0.001**	0.08	0.03	0.19	**<0.001**
**[c] 253,200 cells**	**ß**	**CI-L**	**CI-U**	**P**	**ß**	**CI-L**	**CI-U**	**P**
Reference (Burker)	exp(17)				exp(17)			
StereoCount vs. Burker	58	23	136	**<0.001**	71	23	196	**<0.001**
DNA content vs. Burker	70	25	193	**<0.001**	111	33	384	**<0.001**
StereoCount vs. DNA content	0.73	0.33	2	0.65	0.64	0.2	1.8	0.65
**[d] 349,200 cells**	**ß**	**CI-L**	**CI-U**	**P**	**ß**	**CI-L**	**CI-U**	**P**
Reference (Burker)	exp(21)				exp(21)			
StereoCount vs. Burker	12	4.8	31	**0.001**	21	7	64	**0.016**
DNA content vs. Burker	0.7	0.3	2.0	0.57	7	2	24	**<0.001**
StereoCount vs. DNA content	17	5	50	**0.005**	3.17	0.8	11	0.38

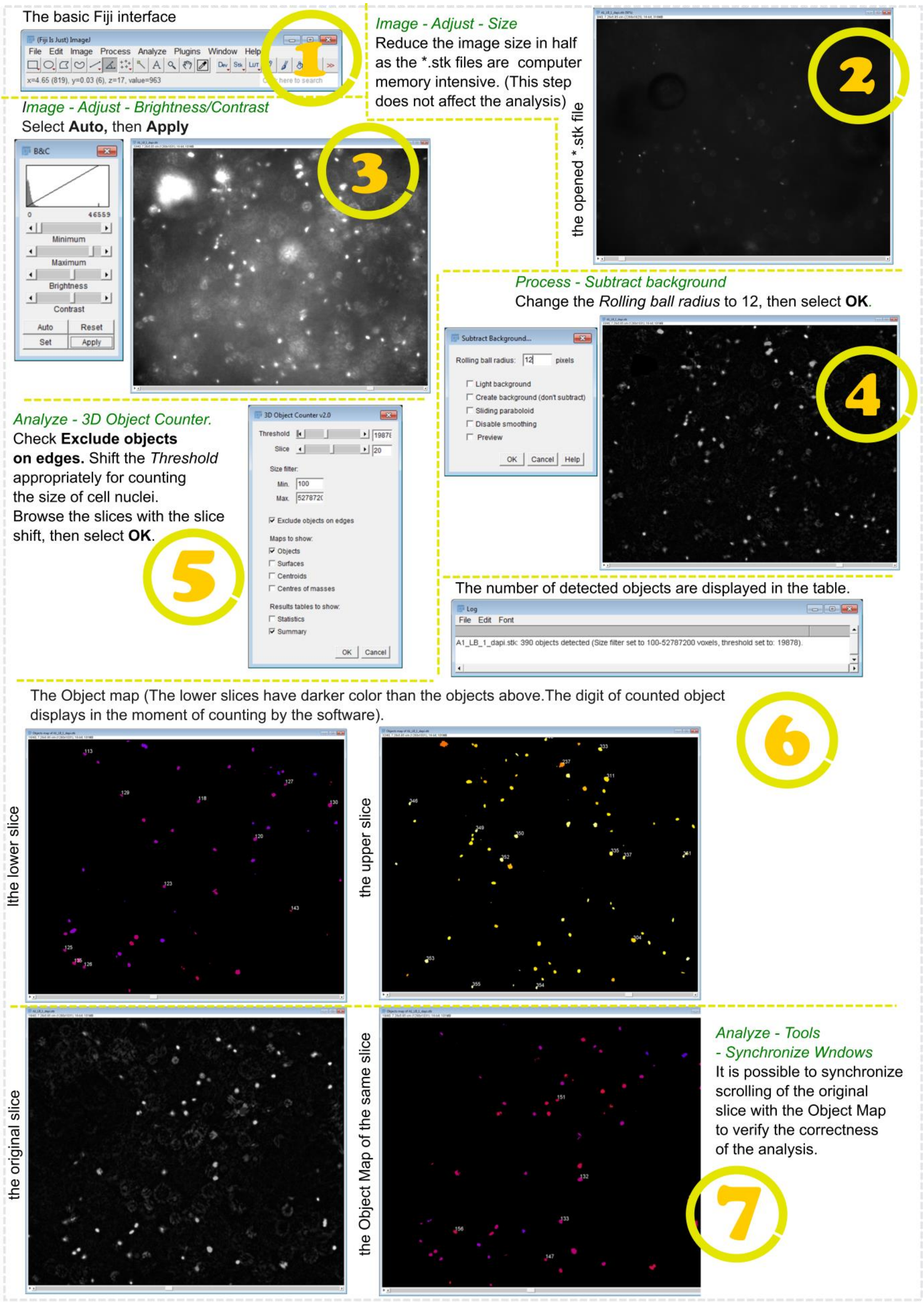

Supplementary Figure S1 Schema of the *StereoCount* data image analysis.

Supplementary Video S1 Homogeneous distribution of viable cells in the collagen scaffold. The cells are stained with Calcein-AM (1µg/ml), *green* (marker of cell viability).
