# Supplementary material for: Novel stereological method for estimation of cell counts in 3D collagen scaffolds
Source: Sci Rep. 2023 May 17;13:7959. doi: 10.1038/s41598-023-35162-z (PMC10192446; doi:10.1038/s41598-023-35162-z)
Supplement: Supplementary file 3 — Supplementary Information 3. [file 41598_2023_35162_MOESM3_ESM.pdf]

```
/* choose input directory, choose output directory
macro changes image to 8bit, creates max intensity projection and
saves in new location
with old name and suffix _MIP

08/2022 Tereza Belinova
Imaging and Optics Facility
Institute of Science and Technology Austria*/

input = getDirectory("Choose directory to process");
output = getDirectory("Choose directory to save images");
list = getFileList(input);
for (f = 0; f < list.length; f++) {
     open(input + list[f]);
     fname = getTitle;
     //deletes original suffix from file name (in our case .stk)
          if( endsWith(fname, ".stk") ){
               name = replace( fname, ".stk", "");
               }
          if(endsWith(fname, ".tif")){
               name = replace( fname, ".tif", "");
               };
     rename("original");
     run("8-bit");
     run("Z Project...", "projection=[Max Intensity]");
     run("Subtract Background...", "rolling=500");
     saveAs("Tiff", output + name +"_MIP.tif");
     close();
     selectImage("original");
     close();
}
showMessage("Job done!");
```
