# Supplementary material for: Novel stereological method for estimation of cell counts in 3D collagen scaffolds
Source: Sci Rep. 2023 May 17;13:7959. doi: 10.1038/s41598-023-35162-z (PMC10192446; doi:10.1038/s41598-023-35162-z)
Supplement: Supplementary file 4 — Supplementary Information 4. [file 41598_2023_35162_MOESM4_ESM.pdf]

```
/*select input and output direcctory
requires masks produced by Labkit segmentation, goes through all
files in directory, detects masked objects
and saves the data as txt file for each folder reporting number of
objects per image

08/2022 Tereza Belinova
Imaging and Optics Facility
Institute of Science and Technology Austria*/

input = getDirectory ("Select folder with masks to be analyzed");
output = getDirectory ("Select folder to save result to");
list = getFileList(input);
savename = getString("Name your output file", "Results of scaffold
XXX")

for (i = 0; i < list.length; i++) {

      //makes sure no ROIs are left
      roiManager("reset");

      //opens listed files
      open(input + list[i]);
      fname = getTitle();
            if( endsWith(fname, ".tif") ){
                  name = replace( fname, ".tif", "");
                  }

      //makes masks visible
      run("Brightness/Contrast...");
      setMinAndMax(0, 0);
      run("Apply LUT");

      //sets treshold default on the mask
      setAutoThreshold("Default dark");
      setOption("BlackBackground", false);
      run("Convert to Mask");
      run("Watershed");

      //analyzes each detected mask ROI
      run("Set Measurements...", "display redirect=None decimal=3");
      run("Analyze Particles...", "size=50-1000 circularity=0.40-1.00
clear summarize add");

}
selectWindow("Summary");

      //saves results as txt file
      saveAs("Results", output + savename + ".txt");
      close("*");

showMessage("Job done. Have a nice day!");
```
